# Effect of a lottery intervention on gender-based violence among female sex workers in Dar es Salaam, Tanzania: results from a randomised trial

**DOI:** 10.1136/bmjph-2025-002587

**Published:** 2026-04-09

**Authors:** Rebecca Hémono, Damien de Walque, Sandra McCoy, Marianna Balampama, William Dow

**Affiliations:** 1 University of California Berkeley School of Public Health, Berkeley, California, USA; 2 World Bank, Washington, District of Columbia, USA; 3 Gilead Sciences, Inc, Foster City, California, USA; 4 Independent Scholar, Dar es Salaam, Tanzania; 5 National Bureau of Economic Research, Cambridge, Massachusetts, USA

**Keywords:** HIV, Epidemiology, Violence

## Abstract

**Introduction:**

Financial incentives are a promising approach for HIV prevention. Some studies have also shown positive spillover effects on gender-based violence (GBV); however, little is known about impacts of financial incentives on GBV among female sex workers (FSW). We investigated the impact of lottery-based incentives on GBV among FSW in Dar es Salaam, Tanzania.

**Methods:**

The RESPECT II study was a parallel-arm trial (n=2206) evaluating the effect of lottery-based incentives on HIV/sexually transmitted infections (STIs) among FSW. Participants were randomised 1:1 to (1) the basic test group (control), including testing and counselling for HIV/STIs and bi-weekly text messages on safe sex or (2) the lottery group, including the basic test group intervention plus entry into a weekly lottery for an award of 100,000 Tanzanian Shilling (US$50) conditional on negative syphilis and *Trichomonas vaginalis* tests. We conducted an intent-to-treat analysis using additive risk models (binomial distribution, identity link) to compare physical and sexual GBV, intimate partner violence (IPV) and non-partner violence between study arms at endline, with estimates expressed as prevalence differences (PDs) and 95% CIs. Causal, population-level impacts were estimated using g-computation.

**Results:**

All forms of violence declined over the study period in the endline sample (n=1117; 49.4% lost to follow-up). There were no significant differences between arms at endline in the primary analysis: GBV – 34.1% control versus 30.7% lottery, PD: −0.03, 95% CI −0.09 to 0.02; IPV – 18.9% control versus 15.9% lottery, PD: −0.03, 95% CI −0.07 to 0.01; non-partner violence – 21.1% control versus 20.4% lottery, PD: −0.01, 95% CI −0.06 to 0.04, nor in the g-computation analysis.

**Conclusion:**

This study did not find any significant impact of low-probability, high-reward lottery incentives on any form of GBV, including IPV and non-partner violence, suggesting that this intervention does not influence risk of violence for FSW in the context of HIV prevention. Nonetheless, given the high attrition in the study sample, effects of the intervention cannot be ruled out, and additional investigation is warranted among a larger sample to further assess the potential benefits and harms of this approach for FSW.

**Trial registration number:**

AEA RCT registry: AEARCTR-0002677.

**WHAT IS ALREADY KNOWN ON THIS TOPIC**

- There is some evidence that suggests financial incentives can mitigate risks of gender-based violence; however, most studies of financial incentives have focused on cash transfers and a paucity of studies have been conducted with female sex workers, who experience disproportionately high risks of violence compared with the general population.

**WHAT THIS STUDY ADDS**

- We examined the impact of a lottery-based incentive intervention on gender-based violence among female sex workers and found no evidence of lottery incentives mitigating or exacerbating experiences of violence when provided as a strategy to motivate safe sex and prevent HIV.

**HOW THIS STUDY MIGHT AFFECT RESEARCH, PRACTICE OR POLICY**

- These results indicate that while financial incentives provided through a low-probability, high-reward lottery mechanism did not reduce risk of gender-based violence for female sex workers, this approach also did not increase risk, suggesting that lottery incentives may be an acceptable approach for reducing risky sexual behaviours. This is important information which can inform programming and policy as investment and interest in incentive mechanisms increases for HIV prevention and care. Nevertheless, further research is warranted with a larger sample, as the high attrition in the study sample cannot rule out the possibility of positive or negative effects.

## Introduction

 Female sex workers (FSWs) are a key population who experience a disproportionately high risk of both HIV and gender-based violence (GBV).^1^,^4^ In sub-Saharan Africa, the prevalence of HIV among FSW is estimated at nearly 30%,^3^ and a study in Tanzania found that more than 50% of FSW had experienced physical or sexual violence in their lifetime.^5^ The interconnected, bidirectional relationship between GBV and HIV is particularly critical for FSW,^6^ who often have limited choices about clients and constrained decision-making before and during sex.^6^,^9^ FSW experiencing GBV are more likely to have higher risk sexual behaviours,^7^ increased HIV incidence^6^ and poor HIV outcomes including suboptimal treatment adherence.^10 11^ In parallel, FSW living with HIV are also at higher risk of GBV, with a study in Tanzania reporting that one-third of HIV-positive FSW had experienced violence in the past 6 months.^10^

In many settings, these sexual and reproductive health risks occur against a backdrop of poverty and economic insecurity, among other structural factors, which serve as primary drivers of sex work.^12^,^14^ Poverty and debt may exacerbate risks of GBV through the creation of additional pressure and urgency to work for sex in order to pay for basic needs such as food and rent.^12 15^ This can create a dynamic where FSWs are not able to decline clients who are known to be violent and thus have heightened vulnerability to coercion and physical and sexual violence, including forced condomless sex, increasing their risk of HIV and other sexually transmitted infections (STIs).^12 16^

Financial incentive approaches have gained increasing attention for their potential to address a wide range of harmful health and social outcomes, including HIV^17^,^19^ and GBV.^20^,^22^ Offering financial incentives has been shown to improve health behaviours via extrinsic motivation^23^ and/or via economic support to address poverty-related barriers influencing health and well-being. While most evidence on incentives to date has focused on conditional cash transfers which offer a guaranteed economic reward contingent on a health behaviour, a broader set of financial incentive mechanisms exists, including those that provide probabilistic rewards, such as lotteries.^24^ The lottery approach similarly aims to motivate positive behaviours through providing an economic reward conditional on behaviour; however, it differs in the predictability of the economic benefit and the extent to which it can be relied on to supplement income.

Because economic security is closely tied to decision-making power and agency for FSW,^9^ it is important to understand how different incentive mechanisms can influence risk of HIV and GBV. We hypothesise that there are several potential pathways by which a lottery-based incentive mechanism could reduce the bidirectional risks of HIV and GBV for FSWs. Most directly, offering the opportunity to win financial rewards conditional on safe sex practices could reduce behaviours that increase risk of STIs or HIV by providing a perceived benefit that would offset the increase in revenue earned for condomless sex. At the same time, the incentive may reduce the risk of client violence, as the reward could motivate FSW to be more selective with clients, avoiding those known to be violent or who present risk behaviours for physical or sexual GBV, such as threats or hostility.^15^ It has also been shown that clients practising condomless sex and other risky sexual behaviours are more likely to perpetrate GBV.^25^ Thus, even if a financial incentive most directly motivates FSW to avoid risky partners to prevent STIs or HIV, it may indirectly have beneficial spillover effects on GBV through reducing contact with clients who are violent. In the context of intimate partner violence, the opportunity to receive a financial incentive could also encourage FSW to avoid or leave violent partners in instances where women stay in violent relationships for financial stability if they perceive the reward as a future pathway to financial agency.^26 27^ Importantly, the probabilistic rewards for this mechanism may be less powerful for credit-constrained FSWs who are in need of immediate income for basic needs. However, it could be more powerful for FSW who are less constrained and do not want to risk losing out on a potentially large reward.^9 28^

Previous evidence shows that even modest expected economic increases can motivate behaviour change,^29^ suggesting that incentives could alter the choices and negotiations FSW undertake during sexual transactions. Furthermore, while lottery incentives do not guarantee income, individuals tend to overweigh small probabilities of large gains, making lottery rewards highly salient.^30^,^32^ This salience may motivate protective behaviours, potentially influencing partner choice, sexual negotiation and the ability to avoid violent clients, even without guaranteed rewards, if the lottery has sufficient perceived value.

Nonetheless, there are two main gaps in the literature: (1) to date, most of the evidence on financial incentives and HIV^33^ and GBV^34^ focuses on cash transfers, limiting generalisability to other incentive mechanisms and (2) these interventions have not been evaluated among FSW, a key population with heightened risks of both GBV and HIV and unique decision-making constraints.^20 35^ While some studies in the general population have shown that cash transfers can effectively decrease physical or sexual violence with intimate partners,^36^,^41^ it is plausible that incentives provided to FSW would not achieve the same impacts, as they offer less financial security than larger cash transfers which can reliably supplement income. On the other hand, the lottery approach could offer similar benefits if it is perceived to be meaningful enough to influence decisions and provide a sense of financial agency, even without guaranteeing income.

We aimed to address this gap by evaluating the effect of a lottery-based incentive mechanism on GBV among FSW participating in the RESPECT II study, a large-scale randomised trial conducted in Dar es Salaam, Tanzania from 2018 to 2022, whose primary goal was to assess the impact of this approach on STIs and HIV incidence. In this analysis, we assessed both IPV and violence perpetrated by non-partners.

## Methods

### Study design, participants and procedures (RESPECT II trial)

The RESPECT II (‘Rewarding STI Prevention and Control in Tanzania’) study was a parallel-arm randomised trial conducted with 2206 FSW in Dar es Salaam, Tanzania from 2018 to 2022 to evaluate the effect of a lottery-based incentive intervention on HIV and STIs (AEA RCT registry: AEARCTR-0002677).^42 43^ Respondent-driven sampling (RDS) was used to identify FSW from various locations (bars, brothels, street) and enrol eligible participants via a coupon system. Ten seeds were selected and were provided with three coded recruitment coupons which were used to recruit participants at sex work venues. FSWs who were enrolled and who completed a baseline survey were given three additional coupons to recruit other peers into the study. The recruitment process continued until the desired sample size was enrolled. Those who helped with recruitment were given 4000 Tanzanian Shillings (TZS) or ~US$2 per participant recruited and enrolled, for up to three participants.

Individuals meeting the following criteria were eligible to participate in the study: (1) female; (2) exchanged sexual intercourse for money in the past 6 months; (3) HIV-negative at enrolment; (4) ≥18 years; (5) not currently pregnant; (6) lived in Dar es Salaam for past 3 months and planned to remain living in Dar es Salaam for at least 2 years following enrolment; (7) had a cell phone able to receive text messages; (8) able to adequately grant informed consent; (9) possessed of a valid coupon (obtained through respondent-driven sampling).

On providing informed consent, FSWs were enrolled by study staff and randomised in a 1:1 ratio to one of two arms using opaque envelopes: (1) the basic test group (control group), which provided testing and counselling for HIV, herpes simplex virus type 2 (HSV2), syphilis and *Trichomonas vaginalis* at baseline and endline, and bi-weekly text messages on safe sex practices; or (2) the lottery group, which provided the basic test group intervention *plus* entry into a random lottery for the opportunity to win a cash prize. In the lottery, 10 FSWs were randomly selected each week for testing of syphilis and *Trichomonas vaginalis*, two curable STIs with reliable, low-cost testing available. Participants who presented for testing were awarded with 100,000 TZS (~US$50) if they received negative test results for both STIs. All participants received free treatment for STIs and counselling if they had a positive test result.

The lottery was designed as a low-probability, high-reward incentive scheme. On average, participants in the lottery group were randomly selected for STI testing one time over the intervention period (24 months) but could receive a large incentive amount of 100 000 TZS each time their name was drawn, conditional on a negative test result. There was a 1% chance of being selected for random testing each week (10 participants selected out of 1110 participants in the lottery arm per week). Given this, the expected value of the incentive was ~1000 TZS per week or ~100 000 TZS over the 104-week intervention period.^43^ This scheme was developed based on theory suggesting that there is preference for a large potential award, even if the chances of winning are low, compared with a guaranteed small award.^30 31^

Baseline data were collected from August 2018 to February 2019. Data collection was paused from March to October 2020 due to COVID-19; endline data collection was completed from June 2021 to January 2022. Surveys were conducted by research assistants from Innovations for Poverty Action Tanzania in Kiswahili on tablets. All surveys were administered in private spaces at a clinic and included an assessment of past 3-month physical and sexual violence.

### Outcomes

In this prespecified secondary analysis of RESPECT II data,^44^ we estimated the effect of the lottery intervention on GBV, expressed as the proportion of FSW who experienced physical and/or sexual violence perpetrated by a spouse, boyfriend or other sexual partner in the past 3 months. This outcome was measured as a binary variable and was assessed through an endline survey at 36 months.

We also investigated the effect of the lottery intervention on two distinct forms of GBV: (1) IPV, defined as physical and/or sexual violence perpetrated by a boyfriend or spouse and (2) physical and/or sexual violence perpetrated by a non-partner (ie, other sexual partners including, but not limited to clients). These forms of GBV are not mutually exclusive and are both components of the GBV indicator described above.

### Sample size and power

The sample size for this study was pre-determined based on the RESPECT II trial, which enrolled 2206 participants at baseline to achieve 80% power to detect a 30 percentage-point (pp) difference in the primary trial outcome of combined HIV/HSV2 incidence. In this analysis, after accounting for endline attrition and baseline reports of past 3-month experiences of GBV, we estimated that we would have 80% power (type I error rate of 5%) for a minimum detectable effect of the lottery intervention on GBV of at least 8 pp.

### Statistical analysis

Analyses were conducted in R V.4.04.^45^ Per our prespecified analysis plan, we descriptively explored sociodemographic characteristics and tested for baseline imbalances between study arms using χ^2^ and t-tests. An intent-to-treat analysis was conducted expressing differences in endline violence between study arms as unadjusted prevalence differences (PDs) with 95% CIs using an additive risk model (binomial distribution, identity link). Adjusted estimates were generated to control for baseline reports of violence.

We used two approaches to account for missing outcome data for 1089 participants (49.4%) in the sample who were not reached for endline surveys and lost to follow-up: multiple imputation and inverse-probability of censoring weighting (IPCW). Multiple imputation was conducted with 20 iterations and estimates were pooled across datasets. IPCW was conducted with age, marital status, parental status (child vs not) and the duration of time living in Dar es Salaam as predictors for missingness (baseline characteristics which significantly predicted missingness in the trial’s primary endline analysis). Models generated using multiple imputation and IPCW were compared with a complete case analysis (excluding missing outcome data) as a sensitivity analysis. Additionally, we assessed differential attrition by regressing a binary indicator of loss to follow-up on baseline characteristics, study arm and their interactions and conducted a joint test of the interactions to evaluate whether attrition patterns differed across study arms.

We also used g-computation to estimate the causal, population-level impact of being randomised to the random test group on violence. This analysis estimates how violence would be affected if everyone in the study population were to have received the lottery-based incentive intervention versus no intervention. Marginal PDs were estimated by setting the exposure for all endline participants (unweighted, complete cases only) to the control group and then to the lottery group, predicting risks for each exposure status, and taking the mean difference of the predicted outcomes; 95% CIs were constructed using bootstrapping.

### Patient and public involvement

FSWs were involved in the RDS sampling procedures to recruit peers for the study.

## Results

**Figure 1:**
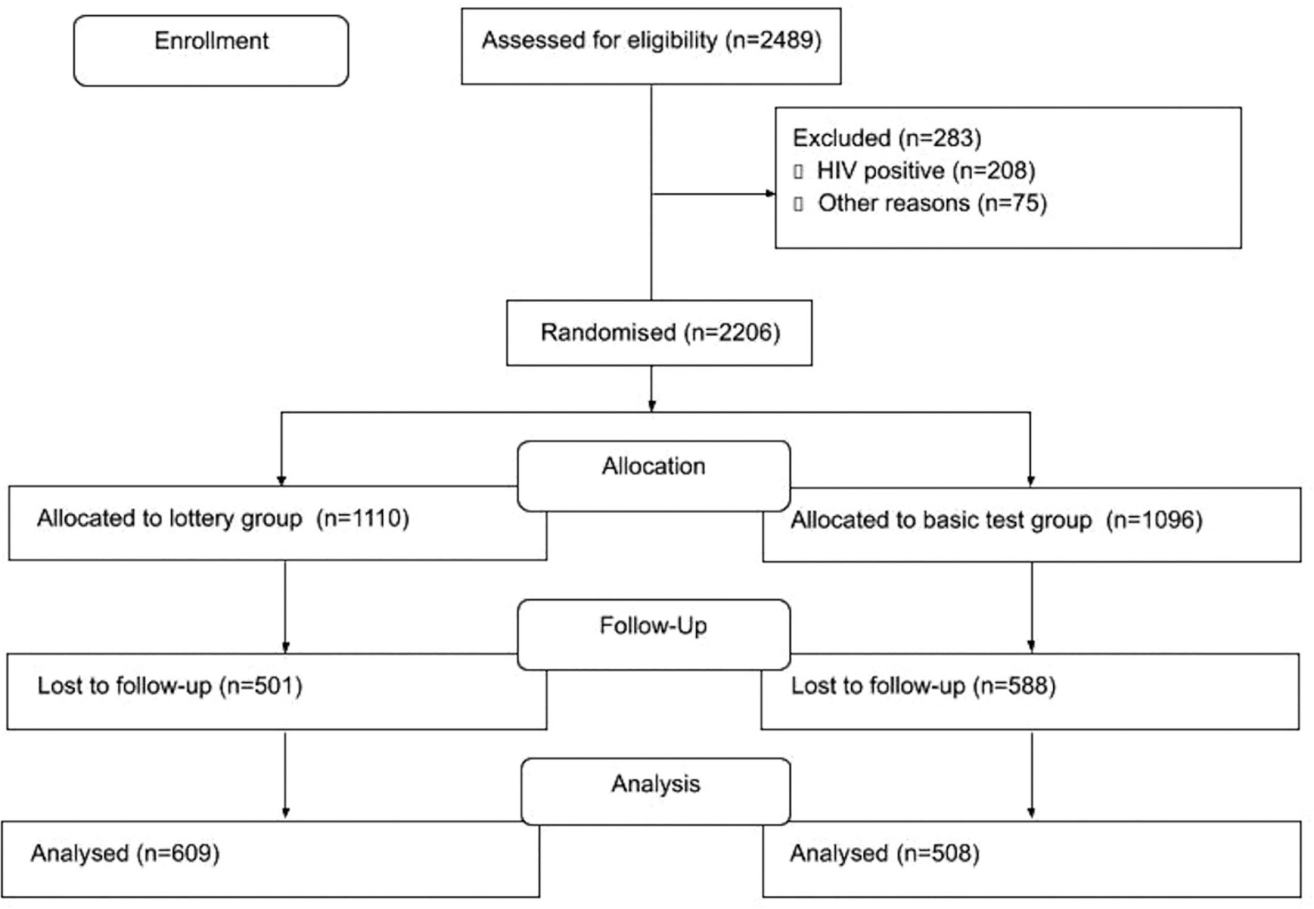
Trial profile.

A total of 2489 individuals were approached for inclusion between August 2018 and February 2019 and 2206 were enrolled in the trial and randomised (figure 1). Of those enrolled, 1117 participants (50.6%) completed endline surveys. The remaining 1089 participants (49.4%) could not be located using their contact information or through inperson visits and were determined to be lost to follow-up.

Table 1 presents the characteristics of study participants at baseline by study arm. The mean age of participants was 26 years and 89.7% had completed primary school or higher. The majority (97.8%) were not partnered or cohabiting; 70.6% reported having children. Sex work was the most common source of income (98.0% of participants) and US$120 was the mean monthly income. There were no meaningful differences in baseline sociodemographic characteristics across the treatment arms.^43^

**Table 1:** Baseline characteristics of study participants in the RESPECT II trial

	Control (n=1096)	Treatment (n=1110)	Overall (n=2206)
Age			
Mean±SD	26±6.6	27±6.8	26±6.7
Median (IQR)	25 (21–30)	25 (21–30)	25 (21–30)
Highest level of education			
None	38 (3.5%)	42 (3.8%)	80 (3.6%)
Some primary	69 (6.3%)	77 (6.9%)	146 (6.6%)
Primary complete	652 (59.5%)	648 (58.4%)	1300 (58.9%)
More than primary	337 (30.7%)	343 (30.9%)	680 (30.8%)
Marital status			
Married/cohabitating	22 (2.0%)	26 (2.3%)	48 (2.2%)
Non-partnered	1074 (98.0%)	1083 (97.6%)	2157 (97.8%)
Has child(ren)	774 (70.6%)	784 (70.6%)	1558 (70.6%)
Number of children*			
Mean±SD	1.6±0.91	1.7±1.1	1.7±0.99
Median (IQR)	1.0 (1.0–2.0)	1.0 (1.0–2.0)	1.0 (1.0–2.0)
Main source of income			
Exchanging sex for money	1083 (98.8%)	1079 (97.2%)	2162 (98.0%)
Other	13 (1.2%)	26 (2.3%)	39 (1.8%)
Monthly income (US$)			
Mean±SD	120±150	120±130	120±140
Median (IQR)	87 (52–150)	87 (52–170)	87 (52–160)
GBV overall*	458 (41.8%)	473 (42.6%)	931 (42.2%)
Physical	379 (34.6%)	385 (34.7%)	764 (34.6%)
Sexual	262 (23.9%)	270 (24.3%)	532 (24.1%)
IPV overall*	213 (19.4%)	204 (18.4%)	417 (18.9%)
Physical	156 (14.2%)	160 (14.4%)	316 (14.3%)
Sexual	125 (11.4%)	120 (10.8%)	245 (11.1%)
Non-partner violence overall*	384 (35.0%)	392 (35.3%)	776 (35.2%)
Physical	308 (28.1%)	319 (28.7%)	627 (28.4%)
Sexual	214 (19.5%)	224 (20.2%)	438 (19.9%)

* Reported in past 3 months.

GBV, gender-based violence; IPV, intimate partner violence.

Sociodemographic characteristics were also similar between the sample retained at endline and those who were lost to follow-up; however, endline participants were slightly older in age, were more likely to have children and had more children than those who were lost to follow-up (online supplemental table 1). A joint test of interactions between study arm and baseline characteristics indicated that attrition patterns did not significantly differ across study arms (p=0.21).

The baseline prevalence of past 3-month GBV overall was 42.2%. Non-partner violence (35.2%) was more common than IPV (18.9%). Physical violence was the most frequently reported type of GBV (34.6%), IPV (14.3%) and non-partner violence (28.4%).

**Figure 2:**
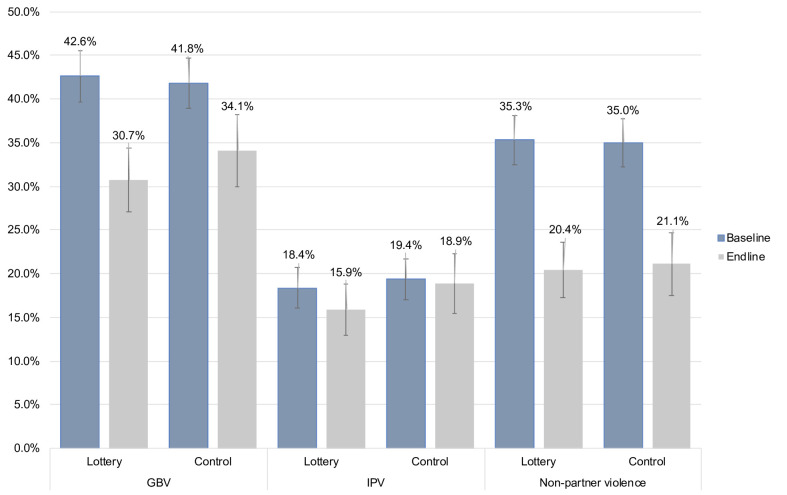
Prevalence (and 95% CIs) of past 3-month violence over the study period (2018–2022), by study arm. GBV, gender-based violence; IPV, intimate partner violence.

Violence declined across all forms of violence in both study arms over the study period; however, decreases were larger in the lottery group than in the control group (figure 2). Overall, the prevalence of GBV declined by 7.7 pp in the control group and 11.9 pp in the lottery group. The prevalence of IPV declined by 0.5 pp in the control group and 2.5 pp in the lottery group; the largest decreases were observed with non-partner violence, which declined by 13.9 pp in the control group and 14.9 pp in the lottery group.

The lottery group also had a lower prevalence of all forms of violence at endline compared with control (table 2). In the complete case analysis, GBV overall and IPV were 3 pp lower in the lottery group than in the control group, and there was a 1 pp difference in non-partner violence between study arms. However, in all models (unadjusted and adjusted complete case analyses and after accounting for missingness), the differences in violence were not statistically significant (table 2, online supplemental table 2).

**Table 2:** Effects of the RESPECT II lottery intervention on past 3-month gender-based violence, intimate partner violence and non-partner violence among FSW at 36 months

	Control (n=508)	Lottery (n=609)	Complete case PD (95% CI)	Multiple imputation PD (95% CI)	IPCW PD (95% CI)	G-computation PD (95% CI)
GBV*	173 (34.1%)	187 (30.7%)	−0.03 (−0.09 to 0.02)	−0.04 (−0.09 to 0.02)	−0.04 (−0.09 to 0.02)	−0.03 (−0.08 to 0.18)
IPV*	96 (18.9%)	97 (15.9%)	−0.03 (−0.07 to 0.01)	−0.03 (−0.08 to 0.03)	−0.04 (−0.08 to 0.01)	−0.03 (−0.08 to 0.01)
Non-partner violence*	107 (21.1%)	124 (20.4%)	−0.01 (−0.06 to 0.04)	0.00 (−0.05 to 0.05)	−0.01 (−0.06 to 0.04)	−0.01 (−0.06 to 0.04)

Data presented as frequency and %; unadjusted PDs with 95% CI generated using generalised linear models with binomial family and identity link.

* Reported in past 3 months.

FSW, female sex worker; GBV, gender-based violence; IPCW, inverse-probability of censoring weighting; IPV, intimate partner violence; PD, prevalence difference.

The predicted marginal prevalence estimated using g-computation was consistent with the observed conditional prevalence. Population-level estimates of GBV overall, IPV and non-partner violence were modestly lower in the intervention group (GBV and IPV: 3 pp lower in lottery group vs control; non-partner violence: 1 pp lower in lottery vs control); however, the width of the CIs indicates no statistical differences between study arms.

## Discussion

This study investigated the impact of a low-probability, high-reward lottery incentive intervention on violence outcomes among FSW in Dar es Salaam, Tanzania to explore whether this financial incentive mechanism mitigates or exacerbates risks of GBV, if at all. We found that at baseline, more than 40% of FSW had experienced physical and/or sexual violence overall (19% IPV; 35% non-partner violence) in the past 3 months. We observed declines in all forms of violence in both study arms at the end of the intervention period. Although there was a modestly lower prevalence of GBV overall, IPV, and non-partner violence in the lottery group compared with control, there were no significant differences between study arms, indicating no effect of the lottery-based incentive on violence outcomes in this population.

While there is mounting evidence that financial incentives can decrease violence against women,^3738 40 46^,^49^ to our knowledge, this is the first study to date to examine a lottery-based incentive and to assess whether a low-probability, high-reward incentive scheme can reduce violence among a key population that experiences high risk of GBV.^1 2 4 50^ Our findings are consistent with some studies conducted in the general population which have shown that cash transfers do not affect GBV.^51 52^ Yet, while we did not find evidence of any beneficial effects of the lottery-based incentive on violence, we also did not observe any harmful effects, as has also been found in some studies in other populations.^37 46 51^ These results underscore that financial incentives have heterogeneous effects on violence depending on the study population and incentive approach and therefore should be examined carefully to ensure the safety and effectiveness in each beneficiary population before implementation.

Our baseline results also reinforce that FSWs experience an alarmingly high burden of violence, as demonstrated in other studies.^1 2 4 50^ Notably, endline participants reported decreases in all forms of violence over the study period, particularly non-partner violence, which decreased by approximately 40% in both study arms. This finding is inconsistent with the existing literature on GBV during COVID-19 indicating that domestic violence increased since the onset of the pandemic,^53 54^ although most previous studies investigated the acute effects of the pandemic on GBV during the initial lockdown/confinement period in 2020 and focused on IPV specifically, which limits their comparability to our finding in this study. In the context of FSW, there may have been a unique period effect, whereby changing social dynamics during the COVID-19 pandemic could have limited interactions with violent perpetrators including clients and reduced the prevalence of GBV. However, there are few rigorous studies which have examined the relationship between COVID-19 and GBV,^55^ thus additional research is needed to better understand the effects of the protracted pandemic on all forms of violence, especially among individuals at high risk of non-partner violence such as FSW. While it is also possible that the observed decreases in GBV could be related to attrition in the study sample (ie, women experiencing violence may have been more likely to be lost to follow-up), the baseline prevalence of violence was similar among endline participants and those who were lost to follow-up, which increases confidence that our results were not driven by preferential attrition related to GBV.

The high attrition in the sample was a critical limitation of this study and had important implications for our analysis.^43^ The RESPECT II trial was designed and powered for the primary outcome of combined incidence of HSV2 and HIV; with the sample size of the parent trial, this analysis was not powered to detect modest GBV effects of less than 8 pp. The substantially reduced endline sample size, with approximately half of all participants lost to follow-up at 36 months, further limited our power and our ability to detect an effect. Attrition may have been due to high mobility in the FSW population^56^ and contextual factors relating to COVID-19, including heightened migration and study delays/longer follow-up periods. Difficulties reaching participants at endline were exacerbated by changing government regulations in Tanzania which required all mobile phone numbers to be registered using biometrics (fingerprints) and national ID cards; those who did not register lost access to their phone number, which was our main mode of contact with study participants. We used multiple imputation and IPCW to account for missing outcomes; however, these techniques may not be sufficient or appropriate for such a high level of missingness, and it is possible that the assumptions required for these approaches were not met (ie, participants were missing at random). Nevertheless, a joint test of interactions between study arm and baseline characteristics indicated that patterns of loss to follow-up did not systematically differ across arms.

Other limitations include the GBV assessment included in the survey, which did not examine violence specifically perpetrated by clients (only ‘other sexual partners’). This impeded our ability to isolate client violence to determine whether there were reductions with this important subgroup as a result of the intervention and to explore our hypothesis that FSW may be more likely to decline clients known to be violent if they had the opportunity to receive a large sum of money elsewhere and did not need to depend on them for income. Finally, this study investigated the lottery-based incentive scheme among FSW only and has limited generalisability to the general population or other key populations that may be beneficiaries of conditional financial incentive programmes.

Despite these limitations, this study fills an important gap in the literature of economic interventions and GBV. Our study sought to understand how lottery-based incentives uniquely affect experiences of violence for FSW. The relationship between different financial incentive schemes, such as cash transfers and GBV has been widely studied. However, no previous studies have examined lottery-based incentives specifically, which could motivate participants differently than other approaches, as lotteries are less reliable, however, offer a higher award if received. Few studies on financial incentives have also been conducted with key populations such as FSW, who experience high risk of both HIV and GBV. Using a randomised design with an endline sample of more than 1110 individuals, this study remains the first of its kind and size to explore this critical question about the effects of lottery-based incentives on violence for FSW.

Improving our understanding of financial incentives for FSW is particularly pertinent as lottery-based incentives have been shown to have positive impacts on other important sexual and reproductive health outcomes such as HIV^57 58^ and STI^59^ testing and prevention, and there is increased interest and investment in financial incentives for reducing risky sexual behaviours and decreasing HIV incidence,^60^ particularly for vulnerable groups who have a high prevalence of GBV such as FSW. Incentives are emerging as a promising approach to address HIV/STIs, and our findings build on this evidence to suggest that lottery-based incentives may not create any unintended harms of violence in the context of HIV/STI prevention for FSW. However, our results also did not show any potential benefits of lottery incentives on GBV. This might be due to the design of the lottery as a low-probability, high-reward incentive scheme, which did not guarantee consistent economic support and improve financial agency or decision-making, as a regular conditional cash transfer might have.

Given the high loss to follow-up at endline, additional investigation is warranted to further assess the effects of this approach for FSW. Enhanced retention procedures, such as incentive-based tracing, may be beneficial for maintaining study participation among this population. Future studies might also explore the lottery-GBV relationship among women in the general population and examine whether this economic approach has differential impacts depending on the regularity, incentive amount and probability of receiving the award.

## Supplementary material

10.1136/bmjph-2025-002587 online supplemental file 1

## Data Availability

Data are available upon reasonable request.
