# Supplementary material for: Effect of a lottery intervention on gender-based violence among female sex workers in Dar es Salaam, Tanzania: results from a randomised trial
Source: BMJ Public Health. 2026 Apr 9;4(2):e002587. doi: 10.1136/bmjph-2025-002587 (PMC13084954; doi:10.1136/bmjph-2025-002587)
Supplement: online supplemental file 1 [file bmjph-4-2-s001.docx]

**Supplemental Table 1. Baseline characteristics comparing participants who participated in endline vs. lost to follow-up**

|  | **Control** | | **Treatment** | | **Overall** | |  |
| --- | --- | --- | --- | --- | --- | --- | --- |
|  | **Lost to follow-up**  **(n=588)** | **Endline sample**  **(n=508)** | **Lost to follow-up**  **(n=501)** | **Endline sample**  **(n=609)** | **Lost to follow-up (n=1089)** | **Endline sample**  **(n=1117)** | **p-value**  **(comparing overall*)** |
| **Age** |  |  |  |  |  |  | 0.001 |
| Mean ± SD | 25 ± 6.2 | 28 ± 6.9 | 25 ± 6.0 | 28 ± 7.3 | 25 ± 6.1 | 28 ± 7.1 |  |
| Median (IQR) | 24 (21, 28) | 26 (22, 32) | 24 (21, 28) | 25 (22, 32) | 24 (21, 28) | 26 (22, 32) |  |
| **Highest level of education** |  |  |  |  |  |  | 0.559 |
| None | 15 (2.6%) | 23 (4.5%) | 20 (4.0%) | 22 (3.6%) | 35 (3.2%) | 45 (4.0%) |  |
| Some primary | 39 (6.6%) | 30 (5.9%) | 30 (6.0%) | 47 (7.7%) | 69 (6.3%) | 77 (6.9%) |  |
| Primary complete | 341 (58.0%) | 311 (61.2%) | 297 (59.3%) | 351 (57.6%) | 638 (58.6%) | 662 (59.3%) |  |
| More than primary | 193 (32.8%) | 144 (28.3%) | 154 (30.7%) | 189 (31.0%) | 347 (31.9%) | 333 (29.8%) |  |
| **Marital status** |  |  |  |  |  |  | 0.725 |
| Married  /cohabitating | 11 (1.9%) | 11 (2.2%) | 11 (2.2%) | 15 (2.5%) | 22 (2.0%) | 26 (2.3%) |  |
| Non-partnered | 577 (98.1%) | 497 (97.8%) | 490 (97.8%) | 593 (97.4%) | 1067 (98.0%) | 1090 (97.6%) |  |
| **Has child(ren)** | 385 (65.5%) | 389 (76.6%) | 342 (68.3%) | 442 (72.6%) | 727 (66.8%) | 831 (74.4%) | <0.001 |
| **Number of children** |  |  |  |  |  |  |  |
| Mean ± SD | 1.5 ± 0.81 | 1.7 ± 0.99 | 1.7 ± 1.1 | 1.8 ± 1.0 | 1.6 ± 0.96 | 1.8 ± 1.0 | 0.001 |
| Median (IQR) | 1.0 (1.0, 2.0) | 1.0 (1.0, 2.0) | 1.0 (1.0, 2.0) | 1.0 (1.0, 2.0) | 1.0 (1.0, 2.0) | 1.0 (1.0, 2.0) |  |
| **Main source of income** |  |  |  |  |  |  | 0.372 |
| Exchanging sex for money | 580 (98.6%) | 503 (99.0%) | 491 (98.0%) | 588 (96.6%) | 1071 (98.3%) | 1091 (97.7%) |  |
| Other | 8 (1.4%) | 5 (1.0%) | 8 (1.6%) | 18 (3.0%) | 16 (1.5%) | 23 (2.1%) |  |
| **Monthly income (USD)** |  |  |  |  |  |  | 0.569 |
| Mean ± SD | 120 ± 180 | 120 ± 110 | 110 ± 110 | 120 ± 150 | 120 ± 150 | 120 ± 130 |  |
| Median (IQR) | 87 (52, 170) | 87 (52, 150) | 87 (52, 150) | 96 (57, 170) | 87 (52, 150) | 87 (56, 160) |  |
| **GBV** | 252 (42.9%) | 206 (40.6%) | 207 (41.3%) | 266 (43.7%) | 459 (42.1%) | 472 (42.3%) | 0.994 |
| Physical | 206 (35.0%) | 173 (34.1%) | 172 (34.3%) | 213 (35.0%) | 378 (34.7%) | 386 (34.6%) | 0.975 |
| Sexual | 142 (24.1%) | 120 (23.6%) | 115 (23.0%) | 155 (25.5%) | 257 (23.6%) | 275 (24.6%) | 0.610 |
| **IPV** | 118 (20.1%) | 95 (18.7%) | 97 (19.4%) | 107 (17.6%) | 215 (19.7%) | 202 (18.1%) | 0.347 |
| Physical IPV | 86 (14.6%) | 70 (13.8%) | 76 (15.2%) | 84 (13.8%) | 162 (14.9%) | 154 (13.8%) | 0.503 |
| Sexual IPV | 69 (11.7%) | 56 (11.0%) | 55 (11.0%) | 65 (10.7%) | 124 (11.4%) | 121 (10.8%) | 0.729 |
| **Non-partner violence** | 214 (36.4%) | 170 (33.5%) | 167 (33.3%) | 225 (36.9%) | 381 (35.0%) | 395 (35.4%) | 0.888 |
| Physical | 168 (28.6%) | 140 (27.6%) | 141 (28.1%) | 178 (29.2%) | 309 (28.4%) | 318 (28.5%) | 0.998 |
| Sexual | 119 (20.2%) | 95 (18.7%) | 93 (18.6%) | 131 (21.5%) | 212 (19.5%) | 226 (20.2%) | 0.691 |
| Missing data: marital status (n=1), main source of income (n=3)  *p-value comparing all participants who were lost to follow-up vs. in the endline sample | | | | | | |  |

**Supplemental Table 2. Adjusted effects of the RESPECT II lottery intervention on gender-based violence, intimate partner violence, and non-partner violence at 36 months, controlling for baseline reports of violence**

|  | **Control**  **(n=508)** | **Lottery**  **(n=609)** | **Complete case**  **PD (95% CI)** | **Multiple imputation**  **PD (95% CI)** | **IPCW**  **PD (95% CI)** | **G-computation**  **PD (95% CI)** |
| --- | --- | --- | --- | --- | --- | --- |
| **GBV** | 173 (34.1%) | 187 (30.7%) | -0.04 (-0.09, 0.02) | -0.04 (-0.10, 0.01) | -0.03 (-0.09, 0.02) | -0.04 (-0.08, 0.01) |
| **IPV** | 96 (18.9%) | 97 (15.9%) | -0.03 (-0.07, 0.02) | -0.03 (-0.08, 0.01) | -0.04 (-0.08, 0.01) | -0.03 (-0.07, 0.02) |
| **Non-partner violence** | 107 (21.1%) | 124 (20.4%) | -0.01 (-0.06, 0.03) | 0.01 (-0.06, 0.03) | -0.01 (-0.05, 0.04) | -0.01 (-0.06, 0.03) |
| Data presented as frequency and %; adjusted prevalence differences with 95% CI generated using generalized linear models with binomial family and identity link, controlling for baseline reports of violence | | | | | | |
